# Connection to Nature and Psychological Wellbeing: The Role of Mindfulness and Spirituality

**DOI:** 10.3390/ijerph23060725

**Published:** 2026-05-29

**Authors:** Simin Kazemi, Julia C. Torquati, Tuyen Huynh

**Affiliations:** 1 Department of Child, Youth, and Family Studies, University of Nebraska Lincoln, 840 N 14th St., 330 Carolyn Pope Edwards Hall, Lincoln, NE 68588, USA; jtorquati@unl.edu; 2 Department of Psychology, Barnwell College, University of South Carolina, 1512 Pendleton Street, Suite #517, Columbia, SC 29208, USA; tuyenh@mailbox.sc.edu

**Keywords:** connection to nature, mindfulness, spirituality, psychological wellbeing

## Abstract

**Highlights:**

**Public health relevance—How does this work relate to a public health issue?**

- Although the mental health benefits of connection to nature are well-documented, public health initiatives lack clarity on the specific psychological mechanisms that translate nature connection into improved wellbeing.
- This research addresses the rising global challenge of human–nature disconnection by investigating how it relates to mindfulness and spirituality, which in turn contribute to psychological wellbeing.

**Public health significance—Why is this work of significance to public health?**

- Across two studies, we show that mindfulness and spirituality serve as pathways linking connection to nature to reduced depression, anxiety, and perceived stress, as well as increased life satisfaction and positive states of mind.
- The impact of these pathways varies depending on how mindfulness and spirituality are conceptualized and measured. Moreover, different facets of mindfulness (e.g., attention vs. acceptance) affect psychological wellbeing in distinct ways.

**Public health implications—What are the key implications or messages for practitioners, policy makers and/or researchers in public health?**

- Practitioners and policymakers should move beyond providing access to nature to fostering a deeper “quality of presence” in nature that nurtures mindful awareness and spiritual relatedness.
- For researchers, the findings underscore the importance of conceptual clarity, as broad or inconsistent measures of mindfulness and spirituality may obscure their role in nature-based mental health interventions.

**Abstract:**

This study examined mindfulness and spirituality as potential explanatory mechanisms underlying the associations between connection to nature and young adults’ psychological wellbeing (depression, anxiety, perceived stress, life satisfaction, and positive states of mind). Two studies employed structural equation modeling (SEM) to test these pathways using different conceptualizations and measures of mindfulness and spirituality. Participants (Study 1: *N* = 219, 69.4% female; Study 2: *N* = 180, 75% female) completed self-report measures of connection to nature, mindfulness, spirituality, and psychological wellbeing. In Study 1, mindful attention functioned as a significant mediating mechanism reducing anxiety and perceived stress, while mindful awareness mediated reductions in depression and increases in positive states of mind. Conversely, spirituality (life scheme and self-efficacy) was not a significant mediator. In Study 2, using alternative measures, spirituality (self-transcendence) significantly operated as a mediating mechanism across all wellbeing outcomes except anxiety, whereas none of the five facets of mindfulness acted as significant mediating mechanisms. Overall, these findings suggest that the roles of mindfulness and spirituality in linking connection to nature and psychological wellbeing may vary depending on how these constructs are conceptualized and measured, highlighting the need for greater conceptual clarity in future research.

## 1. Introduction

A substantial body of interdisciplinary evidence has documented the benefits of nature contact for human health and wellbeing [1,2,3,4,5]. Connection to nature, known as a subjective sense of relatedness and community with nature [6,7] appears to be of particular importance for mental health and wellbeing [5]. Extant research links connection to nature to several dimensions of wellbeing [7,8,9,10] with meta-analytic evidence indicating moderate to large effect sizes across psychological and emotional wellbeing outcomes [2,11]. However, research on the factors or processes that may help explain how connection to nature promotes psychological wellbeing has been limited.

This study addresses this gap by investigating mindfulness and spirituality as potential processes explaining the link between connection to nature and psychological wellbeing. In contrast to a direct association, a mediating mechanism represents an explanatory pathway through which an initial variable (connection to nature) may influence the outcome (psychological wellbeing), thereby clarifying how connection to nature yields health benefits. Given the distinctive qualities of nature, these constructs are theoretically relevant. Nature can evoke a sense of fascination which promotes presence, absorption, and awareness of one’s surroundings—core components of mindfulness. Nature also elicits a feeling of relatedness to something grand which can be a catalyst for spiritual experiences and a deeper sense of purpose [12]. Taken together, these perspectives suggest that mindfulness and spirituality may serve as pathways through which connection to nature contributes to enhanced psychological wellbeing. While mindfulness and spirituality have been independently linked to connection to nature [12,13,14] and to psychological wellbeing [15,16], their role as explanatory pathways remain largely unexamined—a gap the current study aims to bridge. The following sections review the conceptual and empirical intersections among these constructs and outline the hypotheses tested in this study.

### 1.1. Psychological Wellbeing

The World Health Organization (WHO) defines wellbeing as the realization of one’s fullest potential for an optimal physical, psychological, social, spiritual, and economic state of health [17]. One of the most widely used conceptualizations of psychological wellbeing includes purpose in life, positive relationships, autonomy, personal growth, self-acceptance, and environmental mastery [18]. Research examining the link between nature and psychological wellbeing has utilized a diverse range of wellbeing indicators including life satisfaction, purpose and meaning, fulfillment, as well as reduced psychological distress such as stress and anxiety [1]. In the present study, psychological wellbeing was operationalized using measures of depression, anxiety, perceived stress, life satisfaction, and positive states of mind, capturing both positive and negative dimensions of psychological wellbeing.

### 1.2. Connection to Nature

Connection to nature encompasses how people relate to and identify themselves with the natural world and how they perceive their connection with it [19,20]. Connection to nature has been defined as a feeling of deep emotional bond and interconnectedness with nature [21], sense of oneness with nature [6,22], and perception of nature as a community to which humans belong [23]. These conceptualizations encompass thoughts, feelings, and representations of interdependence in associations between self and nature.

### 1.3. Connection to Nature and Psychological Wellbeing

Connection to nature has been associated with emotional, psychological, and social wellbeing [8], life satisfaction [24], flourishing and subjective vitality [25], and sense of purpose in life and self-acceptance [7,26,27]. Nature relatedness was positively associated with happiness, life satisfaction, positive affect, and vitality even after controlling for general connectedness, suggesting a unique psychological contribution of nature relatedness [27]. A meta-analysis of 30 samples yielded a small but significant effect for the association between nature connectedness and positive affect, vitality, and life satisfaction [2]. Positive associations between connection to nature and psychological wellbeing have been documented across several countries and geographic regions such as China [28], Hong Kong [20], the UK [9], Canada [7], Australia [29], Turkiye [30], and the USA [25], providing evidence that the association between connection to nature and psychological wellbeing is a valid and generalized phenomenon.

### 1.4. Mindfulness

Mindfulness is an attention-related psychological construct that refers to present-centered awareness and a nonjudgmental approach to the current moment [31]. Mindfulness has been operationalized in different ways and it is widely recognized as multidimensional, with common elements related to attention—actively sustaining attention to the current experience; awareness—observing both pleasant and unpleasant internal (emotions/thoughts) and external (environment) present experience; non-judging—actively adopting a non-evaluative perspective on the present experience; and acceptance—embodying an attitude of openness and non-reactivity toward the present moment [32,33,34,35].

#### 1.4.1. Mindfulness and Psychological Wellbeing

A robust body of research indicates that trait mindfulness and mindfulness practices (e.g., meditation) can operate as predictors and mediators or moderators of wellbeing [16,36,37]. Empirical evidence indicates mindfulness practices are effective for improving wellbeing among diverse populations, including cancer patients [38,39], veterans with posttraumatic stress disorder (PTSD) [40], children [41,42], and young adults [43]. Mindfulness may promote psychological wellbeing through improved attention [44], reducing stress and decreasing negative affect and depression [45], and promoting positive states of mind [46]. During the COVID-19 pandemic, mindfulness was inversely associated with stress, depression, anxiety, and suicidal thoughts among college students [47].

#### 1.4.2. Connection to Nature and Mindfulness

Research has revealed both direct and mediated associations between connection to nature and mindfulness. A meta-analysis reported a weighted effect size of *r* = 0.25, indicating a moderate positive association between connection to nature and mindfulness. Notably, the strength of this association varied depending on the specific measures of mindfulness used [14]. Connection to nature is positively correlated with mindfulness, reflective self-attention [48] and mindfulness-related facets, such as observing and non-reacting [49]. Emerging research suggests that natural environments may support mindfulness processes, particularly among individuals with limited meditation experience. Mindfulness practices conducted in natural settings have been associated with improved attentional regulation and psychological restoration compared to practices in environments with limited natural stimuli [50,51]. The restorative qualities of nature may help reduce cognitive overload and attentional fatigue, thereby creating conditions that facilitate present-moment awareness and engagement in mindfulness practices. This phenomenon aligns with Attention Restoration Theory, suggesting that inexperienced meditators may benefit from cognitively and physically distancing themselves from everyday environments that often overwhelm attentional resources in order to engage more effectively in mindfulness practices [50].

#### 1.4.3. Connection to Nature, Mindfulness, and Psychological Wellbeing

Connection to nature has been linked to wellbeing through mindfulness. However, the nature and strength of these associations vary depending on the specific facets of mindfulness and how they are conceptualized and measured. A mediation analysis examining the role of five mindfulness facets found partial mediation by non-reactivity and observing in the association between nature relatedness and positive affect, and full mediation of these same facets in the association between nature relatedness and life satisfaction [37]. Howell et al. [8] conducted two studies examining associations among connection to nature, mindfulness, and wellbeing. One study yielded a non-significant association between connection to nature and mindfulness; however, the second study used multiple measures to construct latent factors and reported significant positive associations between connection to nature, mindfulness, and wellbeing. The variability in findings indicates that different mindfulness measures may yield different results, even with the same measures of connection to nature and wellbeing.

### 1.5. Spirituality

Spirituality has been conceptualized and measured in a variety of ways that include affective, cognitive, experiential, and behavioral components. For example, Greenfield et al. defined spirituality as intrapsychic experiences “…that relate to an individual’s sense of connection with something transcendent; integration of the self; and feelings of awe, gratitude, compassion, and forgiveness” [15] (p. 197). Labbé and Fobes defined spirituality as an “orientation toward feeling and/or wanting to be connected to something bigger than oneself” [52] (p. 141). Daaleman and Frey conceptualized spirituality as a combination of meaning or purpose in life (“life scheme”) and self-efficacy [53]. Spirituality has been studied as distinct from religiosity (i.e., engagement in religious practices), as spirituality and religiosity can vary independently. However, one model of spirituality includes religiousness along with a cognitive orientation toward spirituality (i.e., beliefs), an experiential/phenomenological dimension (i.e., mystical and transcendent experience), existential wellbeing (i.e., sense of meaning, purpose and self-efficacy), paranormal beliefs (i.e., belief in paranormal phenomena) [54]. A systematic review of spirituality measures identified transcendence (i.e., connection with something greater than oneself), connectedness (i.e., to a higher power, nature, and other people), and meaning and purpose in life as the most frequent domains included in definitions of spirituality [55].

#### 1.5.1. Spirituality and Psychological Wellbeing

Positive associations between spirituality and diverse indicators of wellbeing have been reported. A meta-analysis of 51 studies of associations between spirituality and quality of life found a moderate effect size, although estimates varied according to measures of spirituality [56]. Greenfield et al. [15] reported that spiritual perceptions, defined as feelings of deep inner peace or harmony, being deeply moved by the beauty of life, strong connection to all life, a sense of deep appreciation, and a profound sense of caring for others, were associated with psychological wellbeing including negative affect, positive affect, purpose in life, positive relations with others, personal growth, self-acceptance, environmental mastery, and autonomy. Similarly, spirituality defined as “inner transcendence” was associated with all of Ryff’s [18] indicators of wellbeing (autonomy, environmental mastery, personal growth, positive relationships with others, purpose in life, and self-acceptance) in a sample of college students [57].

#### 1.5.2. Connection to Nature and Spirituality

Although connection to nature and spirituality have common roots traceable through human history, philosophy, art and literature, their relationship has not been subject to scientific inquiry until recently. Empirical studies examining the association between connection to nature and spirituality offer promising evidence in support of the long-held views regarding the spiritual significance of the natural world [58]. For example, connection to nature has been positively correlated with aspects of spirituality such as transcendence and awe [13,59]. In a cross-cultural study conducted in France and Mexico, connection to nature significantly predicted non-religious spirituality [60].

#### 1.5.3. Connection to Nature, Spirituality, and Psychological Wellbeing

Positive associations between connection to nature, spirituality, and multiple dimensions of wellbeing have been reported [61,62]. For example, Trigwell et al. [26] found that spirituality operationalized as mysticism mediated the relationship between nature connectedness and the wellbeing dimensions of self-acceptance, purpose in life, personal growth, positive relationship with others, and autonomy. However, the mysticism measure included a subscale assessing purpose in life that likely inflated the association with the wellbeing dimension of purpose in life.

### 1.6. The Present Study

Research suggests that mindfulness and spirituality may mediate the association between connection to nature and psychological wellbeing [8,37,62,63,64]; however, they have not been examined as mediators in the same study. This is an important question to consider because there may be overlaps in the operationalization of mindfulness and spirituality that could lead to mis-identifying mediating processes. Additionally, multiple mediating paths are possible. Because findings vary depending upon the measures of mindfulness, spirituality, and psychological wellbeing, we conducted two sequential studies testing a model in which mindfulness and spirituality mediated associations between connection to nature and psychological wellbeing that used the same exogenous and outcome measures but varied the mediating measures across the samples. Connection to nature was assessed using the Connectedness to Nature Scale [6] and psychological wellbeing was operationalized using multiple positive and negative indicators, including depression, anxiety, perceived stress, life satisfaction, and positive states of mind. This approach permitted comparison of findings using different measures of mindfulness and spirituality as mediators. Given that mindfulness is widely defined as nonjudgmental awareness of, attention to, and acceptance of the present moment, we used two measures of mindfulness that emphasized these core elements in Study 1 (The Mindful Attention and Awareness Scale [65], and the Philadelphia Mindfulness Scale [34]). In Study 2, we used the Five Facets Mindfulness Questionnaire [32], which captures a broader range of mindfulness dimensions—describing, observing, acting with awareness, non-judging, and non-reactivity—to allow a more detailed comparison.

Similarly, because transcendence, connectedness, and meaning and purpose in life are the most frequent domains included in definitions and measures of spirituality, we selected a measure that included purpose in life in Study 1 (Spirituality Index of Wellbeing [53]) and a measure of transcendence in Study 2 (Self-Transcendence Measure-Brief [66]). For both studies, we hypothesized that connection to nature would be positively associated with mindfulness and spirituality, which in turn would be inversely associated with depression, anxiety, and perceived stress, and positively associated with life satisfaction and positive states of mind.

## 2. Study 1

### 2.1. Study 1 Methods

#### 2.1.1. Study 1 Participants

Power analysis using G*Power 3.1 [67] indicated that 125 participants are needed to detect an effect size of 0.14 with 80% statistical power (*α* = 0.05) across nine predictors (including covariates). For mediation analysis using bootstrapping, 148 participants are required, assuming small-to-medium effects (*r* = 0.26) for both paths [68].

Undergraduate students (*N* = 219) from a Midwestern university participated in an online survey. We only retained completed survey submissions for analysis. See Table 1 for participants’ demographic information. Most participants were recruited from social science classes. Participants received extra course credit from their instructors upon completion of the survey. The university’s Institutional Review Board approved this study. Participants provided informed consent.

#### 2.1.2. Study 1 Measures

##### Connection to Nature

The Connectedness to Nature Scale measures “affective, experiential connection to nature” [6] (p. 504) and includes 14 items rated on a 5-point scale (1 = “Strongly Disagree” to 5 = “Strongly Agree”). A sample item includes, “I often feel a kinship with animals and plants.” Items 12 (“When I think of my place on Earth, I consider myself to be a top member of a hierarchy that exists in nature.”) and 14 (“My personal welfare is independent of the welfare of the natural world.”) were excluded based on their low item-total correlations and an increase in Cronbach’s alpha if item deleted. The mean score was then calculated using the remaining items (α = 0.82). Higher scores reflected greater connection to nature.

##### Mindfulness

The Mindful Attention Awareness Scale (MAAS [65]; α = 0.84) includes 15 items rated on a 6-point scale (1 = “Almost Always” to 6 = “Almost Never”), assessing awareness and attentiveness to current experiences. A sample question is, “I find it difficult to stay focused on what’s happening in the present.” The mean score was used, with higher scores indicating greater attentiveness to the experience.

Philadelphia Mindfulness Scale (PHLMS) [34] is a 20-item instrument with two 10-item subscales assessing mindful awareness (e.g., “I am aware of what thoughts I’m having when my mood changes”; α = 0.77) and mindful acceptance (“There are aspects of myself I don’t want to think about”; α = 0.88) rated on a 5-point scale (1 = “Never” to 5 = “Very Often”). Items for the mindful acceptance subscale were reverse coded, and mean scores for mindful awareness and mindful acceptance were then calculated, with higher scores indicating greater levels of both awareness and acceptance.

##### Spirituality

The Spirituality Index of Wellbeing [53] is a 12-item measure with each statement rated on a 5-point scale (1 = “Strongly Disagree” to 5 = “Strongly Agree”) to assess perceptions of spirituality. Items are divided into two subscales: life scheme (e.g., “I have lack of purpose in my life”; α = 0.91) and self-efficacy (e.g., “I can’t begin to understand my problems”; α = 0.83). Items were reverse coded so that higher scores reflected higher levels of life scheme and self-efficacy. Subsequently, mean scores for life scheme and self-efficacy were calculated.

##### Psychological Wellbeing

The Hospital Anxiety and Depression Scale (HADS) [69] includes 14 items rated on a scale from 0 to 3. Items were divided into two subscales: anxiety (e.g., “I get sudden feelings of panic”; α = 0.83) and depression (e.g., “I feel as if I am slowed down”). A mean score for the anxiety subscale was calculated using all items, with higher scores indicating greater anxiety. Items 10 (“I have lost interest in my appearance.”) and 14 (“I can enjoy a good book or radio or TV program.”) of the HADS depression subscale were excluded due to their low item-total correlation. A mean score for the depression subscale was then calculated using the remaining items (α = 0.72), with higher scores indicating higher depression.

The Perceived Stress Scale (PSS [70]; α = 0.85) is a 10-item measure (e.g., “In the last month, how often have you felt nervous and stressed?”), which assesses one’s thoughts and feelings about a negative event that happened during the past month on a 5-point scale (0 = “Never” to 4 = “Very Often”). The mean score was used, with higher scores indicating higher perceived stress.

The Satisfaction with Life Scale (SWLS [71]; α = 0.89) includes five items (e.g., “The conditions of my life are excellent.”) rated on a 7-point scale (1 = “Strongly Disagree” to 7 = “Strongly Agree”). The mean score was used with higher scores reflecting more satisfaction with life.

The Positive States of Mind Scale (PSOMS [72]; α = 0.83), is a 6-item measure of positive thoughts and feelings regarding events occurring over the past week. Each statement (e.g., “Feeling able to attend to a task you want or need to, without many distractions from within yourself”) is rated on a 3-point scale (0 = “Unable to Have It” to 3 = “Have It Easily”). The mean score was calculated, with higher scores indicating greater positive states of mind.

#### 2.1.3. Study 1 Data Analytic Plan

Path analysis was conducted using Mplus version 8.10 [73]. Full information maximum likelihood (FIML) estimation was used to address missing data [74]. Several indices were used to assess the model fit, including Comparative Fit Index (CFI), Root Mean Square Error of Approximation (RMSEA), and Standard Root Mean Residual (SRMR), with CFI > 0.95 [75,76], RMSEA < 0.06–0.08 [76], and SRMR < 0.08 [75,76] indicating acceptable cutoff values.

A bootstrap approach [77] was used for estimating indirect effects and for addressing non-normality and maximizing power while minimizing Type I error. This approach provides an empirical approximation of the sampling distribution of indirect effects to produce confidence intervals (CIs) of estimates. Bootstrapping can determine if an indirect effect is absent (CI contains zero) or present (CI does not contain zero) [78]. We used a nonparametric resampling method (bias-corrected bootstrap) with 1000 resamples drawn to derive the 95% CIs for the indirect effects [79]. We controlled for gender, race, and year in college (as a proxy for age) in our mediation analysis.

### 2.2. Study 1 Results

#### 2.2.1. Study 1 Pearson Correlations

Descriptive statistics and correlations for all variables are presented in Table 2. The associations between connection to nature and all measures of spirituality and psychological wellbeing were non-significant. All the mediators (mindfulness and spirituality) were significantly correlated with one another and all dimensions of psychological wellbeing, except for mindful awareness. The associations between mindful awareness and mindful acceptance, anxiety, and perceived stress were non-significant.

#### 2.2.2. Study 1 Mediation Analysis

We tested a mediation model in which mindfulness (mindful attention, mindful awareness, and mindful acceptance) and spirituality (life scheme and self-efficacy) mediated the associations between connection to nature and psychological wellbeing. Indicators of mindfulness, spirituality, and psychological wellbeing were independently tested as observed variables in the model. The initial model fit was unacceptable (χ^2^(25) = 295.574, *p* < 0.001; CFI = 0.735; RMSEA = 0.223; SRMR = 0.198). Modification indices suggested that freely estimating the residual correlation between certain indicators of mindfulness and spirituality would improve the model fit, indicating that corresponding indicators shared something unique. This adjustment was conceptually consistent with the fact that these indicators are either the subscales from the same instrument (e.g., self-efficacy and life scheme for spirituality; mindful awareness and acceptance for mindfulness), or measures of the same construct (e.g., MAAS for mindfulness). Guided by the modification indices, and rather than allowing the indicators’ residuals to correlate freely, we respecified the model by introducing latent mindfulness and spirituality constructs to account for the shared variance among their indicators. This approach more appropriately reflects the underlying conceptual structure of the constructs to account for the shared variance, while simultaneously allowing us to examine the unique independent pathways of the individual indicators to the outcome variables. Importantly, these latent constructs were not modeled as mediators themselves; rather, they were specified as a measurement control to partial out shared variance among the indicators, thereby improving overall model fit to acceptable levels. Although the chi-square statistic remained significant (χ^2^(19) = 51.355, *p* < 0.001), alternative fit indices for the respecified model indicated reasonable fit. The respecified model for Study 1 yielded fit indices of CFI = 0.968, RMSEA = 0.088, and SRMR = 0.046. Although the CFI and SRMR met standard acceptable thresholds, the RMSEA fell slightly above the standard <0.08 cutoff value. This suggests a marginal overall model fit, indicating that the structural paths should be interpreted with appropriate caution. However, it is important to note that in models with lower degrees of freedom or smaller sample sizes, RMSEA has been reported to have a tendency to over-reject true models [75].

**Figure 1:**
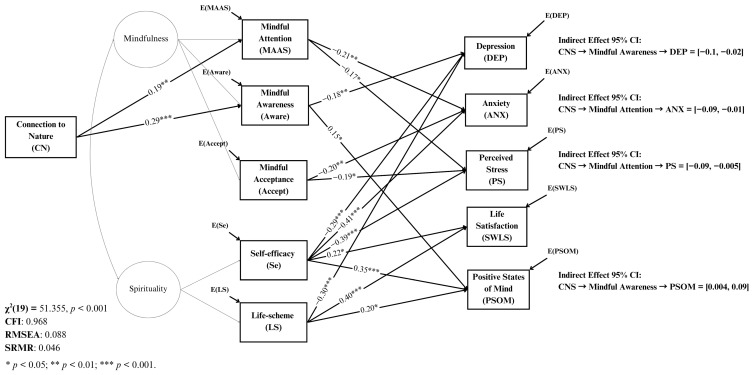
Model of Study 1 showing mindful attention mediating the associations between connection to nature (CN) and both anxiety and perceived stress, and mindful awareness mediating the associations between depression and positive states of mind; standardized coefficients for significant paths and 95% confidence intervals (CIs) for significant indirect effects are reported, along with the Comparative Fit Index (CFI), Root Mean Square Error of Approximation (RMSEA), and Standard Root Mean Residual (SRMR). Rectangles represent observed variables. Ovals represent latent mindfulness and spirituality constructs, depicting and accounting for the shared variance among the observed indicators of mindfulness and spirituality.

A significant chi-square difference test showed that the respecified model with smaller χ^2^ value provided better fit. Therefore, we retained this model (See Figure 1).

The model significantly explained 39% of the variance in depression, 41% of the variance in anxiety, 42% of the variance in perceived stress, 42% of the variance in life satisfaction, and 36% of the variance in positive states of mind.

Mindful attention significantly mediated the association between connection to nature and anxiety *β* = −0.04, 95% CI = [−0.09, −0.01], and perceived stress *β* = −0.03, 95% CI = [−0.09, −0.005], but not other indicators of psychological wellbeing. Mindful awareness significantly mediated the association between connection to nature and depression *β* = −0.05, 95% CI = [−0.1, −0.02], and positive states of mind *β* = 0.04, 95% CI = [0.004, 0.09]. Mindful acceptance significantly predicted anxiety *β* = −0.20, *p* = 0.002, 95% CI = [−0.33, −0.07], and perceived stress *β* = −0.19, *p* = 0.02, 95% CI = [−0.34, −0.03]; however, it did not mediate the association between connection to nature and any of the psychological wellbeing outcomes.

None of the dimensions of spirituality were significant mediators of the association between connection to nature and psychological wellbeing. However, self-efficacy significantly predicted all indicators of psychological wellbeing (depression *β* = −0.29, *p* < 0.001, 95% CI = [−0.45, −0.13]; anxiety *β* = −0.41, *p* < 0.001, 95% CI = [−0.55, −0.26]; perceived stress *β* = −0.39, *p* < 0.001, 95% CI = [−0.54, −0.24]; life satisfaction *β* = 0.22, *p* = 0.01, 95% CI = [0.05, 0.38]; positive states of mind *β* = 0.35, *p* < 0.001, 95% CI = [0.19, 0.53]). Life scheme significantly predicted depression *β* = −0.30, *p* < 0.001, 95% CI = [−0.47, −0.16], life satisfaction *β* = 0.40, *p* < 0.001, 95% CI = [0.24, 0.53], and positive states of mind *β* = 0.20, *p* = 0.02, 95% CI = [0.03, 0.35].

### 2.3. Study 1 Discussion

The findings from Study 1 support the hypothesized mediating role of specific mindfulness facets in the relationship between connection to nature and psychological wellbeing. Specifically, mindful attention (MAAS) mediated the effect of connection to nature on anxiety and perceived stress. In addition, mindful awareness (PHLMS) mediated the associations with depression and positive states of mind. These results align with prior research documenting the associations between connection to nature, mindfulness, and psychological wellbeing [8,25,48]; however, it is important to note that none of these studies examined mediation. Consistent with previous research [8,64] mindful acceptance was not a significant mediator. The varying mediating roles of these mindfulness facets across different indicators of psychological wellbeing highlight the importance of examining mindfulness dimensions separately, as they may relate to connection to nature in distinct ways and exert different effects on psychological wellbeing.

Regarding spirituality, the construct as operationalized via self-efficacy and life scheme, was not a significant mediator. This divergence from previous studies identifying spirituality as a significant pathway [26,62] may be due to measurement differences. These studies utilized measures of mysticism that emphasize the transcendental aspects of spirituality and inner experiences that give meaning to life. The lack of significant mediation in our study may reflect limitations in how spirituality was operationalized, suggesting a need for alternative conceptualization and measurement that better align with aspects of spirituality that are well-established foci of research [55]. Consequently, we extended our investigation in Study 2 to examine whether different operational definitions of mindfulness and spirituality might better capture these mediating processes.

## 3. Study 2

### 3.1. Study 2 Methods

#### 3.1.1. Study 2 Participants

Power analysis using G*Power 3.1 [67] indicated that 130 participants are needed to detect an effect size of 0.14 with 80% statistical power (α = 0.05) across ten predictors (including covariates). To detect mediation effects using a bootstrapping procedure, a sample of 148 is required, assuming a small-to-medium effect size (*r* = 0.26) for both paths [64].

Undergraduate students (*N* = 180) from a Midwestern university participated in an online survey. The online survey utilized a forced-response setting in Qualtrics to prevent item-level missing data, and only completed survey submissions were retained for analysis. See Table 3 for participants’ demographic information. Participants represented various majors, including education, human development and family sciences, psychology, environmental and sustainability studies, exercise and health sciences, nursing, and criminal justice. They received extra course credit from their instructors upon completion of the survey. The study was approved by the university’s Institutional Review Board, and all participants provided informed consent.

#### 3.1.2. Study 2 Measures

##### Connection to Nature and Psychological Wellbeing

Measures for connection to nature and dimensions of psychological wellbeing were consistent with those used in Study 1 in order to examine how different measures of mindfulness and spirituality operate as mediators in models with the same exogenous and outcome variables. To maintain comparability, the same items were excluded from the Connection to Nature Scale and the HADS depression subscale to improve the internal consistency of the measures. Descriptive information and Cronbach’s alphas for these measures are presented in Table 4.

##### Mindfulness

The Five Facet Mindfulness Questionnaire measures the general tendency towards mindfulness in daily life (FFMQ) [32] and includes 39 items rated on a 5-point scale (1 = “never or very rarely true” to 5 = “very often or always true”). Observing subscale contains 8 items (e.g., “I pay attention to sensations, such as the wind in my hair or the sun in my face”; α = 0.75). Describing subscale contains 8 items (e.g., “I can usually describe how I feel at the moment in considerable detail”; α = 0.86). Acting with awareness subscale contains eight items (e.g., “I do jobs or tasks automatically without being aware of what I’m doing”; α = 0.84). Non-judging subscale contains 8 items (e.g., “I tell myself I shouldn’t be feeling the way I’m feeling”; α = 0.88), and the non-reactivity subscale consists of 7 items (e.g., “I perceive my feelings and emotions without having to react to them”; α = 0.71). Acting with awareness, and non-judging subscales were reverse coded, and the mean score was calculated for each facet separately, with higher scores indicating greater levels of that mindfulness facet.

##### Spirituality

The Self-Transcendence Measure-Brief (STM-B [66]; α = 0.86) is a 10-item measure with each statement rated on a 5-point scale (0 = “not at all characteristic of me or my beliefs” to 4 = “a great deal characteristic of me or my beliefs”). A sample item includes “My life is meaningful because I live for something greater than myself.” The mean score was calculated with higher scores reflecting greater levels of spirituality.

#### 3.1.3. Study 2 Data Analytic Plan

The analytical plan for Study 2 paralleled that to Study 1, the only difference was the substitution of the measures of mindfulness and spirituality.

### 3.2. Study 2 Results

#### 3.2.1. Study 2 Pearson Correlations

Descriptive statistics and correlations for all variables are presented in Table 4. Connection to nature was positively correlated with the observing facet of mindfulness as well as spirituality. The associations between connection to nature and all measures of psychological wellbeing were non-significant. Among the facets of mindfulness, the describing dimension was significantly correlated with all other mindfulness facets, spirituality, and all indicators of psychological wellbeing. Observing was only positively correlated with non-reactivity, spirituality, and unexpectedly, positively correlated with anxiety. All facets of mindfulness except observing were significantly correlated with all dimensions of psychological wellbeing. Spirituality was correlated with all dimensions of psychological wellbeing except anxiety.

#### 3.2.2. Study 2 Mediation Analysis

**Figure 2:**
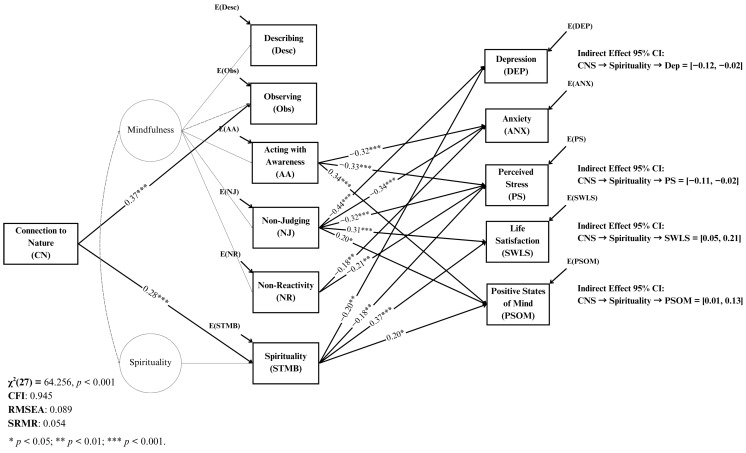
Model of Study 2 showing spirituality mediating the associations between connection to nature (CN) and depression, perceived stress, life satisfaction, and positive states of mind. Standardized coefficients for significant path and 95% confidence intervals (CIs) for significant indirect effects are reported, along with the Comparative Fit Index (CFI), Root Mean Square Error of Approximation (RMSEA), and Standard Root Mean Residual (SRMR). Rectangles represent observed variables. Ovals represent latent mindfulness and spirituality constructs, depicting and accounting for the shared variance among the observed indicators of mindfulness and spirituality.

In Study 2, we tested the same model as in Study 1 in which mindfulness and spirituality mediated the associations between connection to nature and psychological wellbeing. We used different measures of mindfulness and spirituality in Study 2 to examine whether different conceptualization and operationalization of the constructs yielded different results. In this model, observed variables of mindfulness were five facets (indicators) of the same mindfulness instrument (FFMQ), and spirituality was measured using a unidimensional instrument (STM-B). The initial model fit was inadequate (χ^2^(33) = 193.718, *p* < 0.001; CFI = 0.761; RMSEA = 0.167; SRMR = 0.146). With the same justification presented in Study 1, we respecified the model by introducing latent variables for mindfulness and spirituality to reflect the underlying constructs more accurately and also to account for measurement error. This re-specification improved the model fit. Similar to the model in Study1, the chi-square statistic remained significant (χ^2^(27) = 64.256, *p* < 0.001); however, alternative fit indices for the respecified model indicated reasonable fit: CFI = 0.945, RMSEA = 0.089 and SRMR = 0.054 (See Figure 2). Because the RMSEA slightly exceeded the standard cutoff value of <0.08, the structural paths should be interpreted with appropriate caution. However, as noted in Study 1, this elevation is likely a reflection of the RMSEA’s documented tendency to over-reject true models in smaller sample sizes or models with fewer degrees of freedom [75]. The model significantly accounted for 27% of the variance in depression, 47% of the variance in anxiety, 46% of the variance in perceived stress, 36% of the variance in life satisfaction, and 36% of the variance in positive states of mind.

None of the facets of mindfulness were significant mediators of the relationship between connection to nature and indicators of psychological wellbeing. However, non-judging was a significant predictor of all indicators of psychological wellbeing (depression *β* = −0.44, *p* < 0.001, 95% CI = [−0.60, −0.26]; anxiety *β* = −0.34, *p* < 0.001, 95% CI = [−0.51, −0.18]; perceived stress *β* = −0.32, *p* < 0.001, 95% CI = [−0.48, −0.16]; life satisfaction *β* = 0.31, *p* < 0.001, 95% CI = [0.14, 0.46]; positive states of mind *β* = 0.21, *p* = 0.009, 95% CI = [0.05, 0.36]). Acting with awareness was a significant predictor of anxiety *β* = −0.32, *p* < 0.001, 95% CI = [−0.46, −0.19], perceived stress *β* = −0.33, *p* < 0.001, 95% CI = [−0.46, −0.16], and positive states of mind *β* = 0.34, *p* < 0.001, 95% CI = [0.19, 0.48]. Non-reactivity was a significant predictor of anxiety *β* = −0.18, *p* = 0.005, 95% CI = [−0.30, −0.04], and perceived stress *β* = −0.21, *p* = 0.001, 95% CI = [−0.33, −0.08]. Describing and observing didn’t significantly predict any indicators of psychological wellbeing.

Spirituality significantly mediated the association between connection to nature and all indicators of psychological wellbeing except anxiety (depression *β* = −0.06, 95% CI = [−0.12, −0.02]; perceived stress *β* = −0.06, 95% CI = [−0.11, −0.02]; life satisfaction *β* = 0.11, 95% CI = [0.05, 0.21]; positive states of mind *β* = 0.06, 95% CI = [0.01, 0.13]).

### 3.3. Study 2 Discussion

The findings from Study 2 indicated that mindfulness as measured by the Five Facets Mindfulness Questionnaire (FFMQ) was not a significant mediator. In contrast, spirituality measured as self-transcendence significantly mediated the association between connection to nature and all indicators of psychological wellbeing except anxiety—an effect that was not observed in Study 1. The inconsistency in the mediation effects of mindfulness and spirituality across the two studies highlights the critical importance of conceptualization and measurement of these constructs in detecting the mediating effects of how connection to nature impacts psychological wellbeing.

## 4. General Discussion

The purpose of this study was to examine mindfulness and spirituality as mediators of the association between connection to nature and psychological wellbeing. Two studies were conducted using different measures of mindfulness and spirituality to allow comparison across conceptualizations and measurements of these constructs as mediators. Unexpectedly, connection to nature was not significantly correlated with any of the measures of psychological wellbeing, in contrast to previous research with undergraduate students that reported positive associations between connection to nature and social and psychological wellbeing [8]. However, it is possible that the increasing rates of mental health challenges among college students [80] may attenuate associations between connection to nature and wellbeing that were found over 15 years ago. Despite the lack of direct associations, we found significant indirect effects with mindfulness-mediating associations in Study 1 and spirituality-mediating associations in Study 2. In Study 1, mindful attention (MAAS) and awareness (PHLMS) mediated the association between connection to nature and specific psychological wellbeing indicators, whereas spirituality (life scheme and self-efficacy) did not. In Study 2, none of the five mindfulness facets served as mediators; however, spirituality operationalized as self-transcendence significantly mediated associations between connection to nature and depression, perceived stress, life satisfaction, and positive states of mind. Results are discussed in greater detail below with attention to the distinct conceptualizations and measures of mindfulness and spirituality.

### 4.1. Mindfulness as a Mediator

In Study 1, mindful attention (MAAS) [65] was positively correlated with connection to nature and significantly mediated the associations between connection to nature and anxiety and perceived stress. Mindful awareness [34] was also positively correlated with connection to nature and significantly mediated the associations between connection to nature and depression and positive states of mind. In Study 2, none of the five facets of the FFMQ [32] mediated the associations between connection to nature and psychological wellbeing, and only the observing facet was positively correlated with connection to nature. These mixed findings may be partly explained by measurement differences as a meta-analytic review showed that the relationship between connection to nature and mindfulness varied depending on mindfulness measures; however, no such difference was found between studies using different measures of connection to nature [14].

#### 4.1.1. Attention and Awareness

Similarities and differences in the three mindfulness measures used in this study may help explain the different results between the two studies. MAAS operationalizes mindfulness as present moment attentiveness [8,65], and the PHLMS subscale operationalizes mindfulness as awareness of internal and external experiences [34]. The FFMQ observing facet is similarly operationalized as noticing internal experiences (e.g., sensations, thoughts, and emotions) and external experiences (e.g., sights, sounds, and smells [81]). The positive effect of connection to nature on mindful attention and awareness (Study 1) and on observing (Study 2) was consistent across the two studies. The effect of nature on attention is well-documented (see Attention Restoration Theory [82]) and may help explain the observed pathways from connection to nature to reduced anxiety, perceived stress, and depressive symptoms, and greater positive states of mind via mindful attention and awareness. Natural environments may foster mindful attention and awareness, as they evoke “fascination,” less effortful attention that draws interest and curiosity [82]. This outward focus can provide a comfortable context for a person to be present with their thoughts and feelings without draining attentional resources. This process can be mentally restorative, offering a sense of “being away” from daily demands and overstimulating environments [82], which supports increased attention, stress recovery, and ultimately greater psychological wellbeing. However, it is important to note that our study did not measure participants’ exposure to nature but rather focused on their felt connection to nature. Nevertheless, it is likely that a stronger feeling of connection to nature corresponds with greater attentiveness to it.

The non-significant associations between observing and psychological wellbeing in Study 2 also align with prior studies [83,84]. For example, no association or positive association between observing and anxiety in non-meditating populations has been noted in previous research [84]. In our study, while this bivariate correlation was positive and statistically significant (*r* = 0.18, *p* < 0.05), the direct path became non-significant in the full structural model. This relationship may suggest that high levels of observing, particularly when not accompanied by other mindfulness facets, may be associated with heightened anxious arousal [35]. This pattern appears to be more salient in non-meditating populations, indicating that an increased capacity to observe and notice internal and external experiences, without the support of other mindfulness facets that require intentional practice and facilitate a non-evaluative openness to the experience—such as non-judging and non-reactivity—may result in unexpected psychological outcomes, including increased anxiety. This suggests potential interacting effects among mindfulness facets, particularly between observing and facets such as non-judging and non-reactivity, in shaping psychological outcomes [84].

#### 4.1.2. Acceptance

Mindful acceptance measured by the PHLMS [34] emphasizes the nonjudgmental dimensions of mindfulness [31,33]. In Study 1, higher levels of acceptance were associated with lower anxiety and depression. However, we did not find a significant effect of connection to nature on acceptance. Similarly, in Study 2, connection to nature did not predict non-judging and non-reactivity, the two FFMQ facets closest to capturing the accepting, non-evaluative nature of mindfulness. Notably, higher non-reactivity was associated with lower anxiety and depression, and non-judging was a predictor of all indicators of psychological wellbeing in Study 2.

These findings suggest that while feeling connected to nature may be associated with enhanced attentiveness to experiences, it does not necessarily cultivate a greater capacity for acceptance or a nonjudgmental stance [34]. Cultivating acceptance may require more deliberate training and mindfulness practice. This interpretation aligns with Baer et al. [32] finding of a negative correlation between observing and non-judging facets of FFMQ, suggesting that for individuals without meditation experience, being attentive to an experience may be accompanied by evaluative judgment rather than openness. Moreover, heightened awareness or attention without acceptance has sometimes shown detrimental impacts on psychological wellbeing [34]. This highlights the interactive nature of mindfulness dimensions (i.e., changes in one dimension tend to impact changes in other dimensions). This change may be particularly relevant regarding the non-judging/acceptance dimension, as outcomes tend to vary depending on an individual’s capacity for acceptance. This represents an open avenue for further investigation.

### 4.2. Spirituality as a Mediator

In Study 1, spirituality, operationalized as meaning in life and self-efficacy [53], was not significantly associated with connection to nature and was not a mediator of connection to nature and psychological wellbeing. To our knowledge, no previous studies of connection to nature and spirituality have used the Spirituality Index of Wellbeing [53] so we cannot directly compare to prior research. However, this finding differs from studies using other spirituality measures that significantly mediated connection to nature and wellbeing. Sanyer et al. [13] found that connection to nature significantly associated with self-transcendence measured via the Nondual Awareness Dimensional Assessment [85], although self-transcendence was not construed as spirituality in that study. Navarro et al. [60] found that connection to nature was associated with spirituality assessed via the nonreligious explicit spirituality scale, which is only available in French and Spanish so we cannot make a direct comparison of findings. We chose the Spirituality Index of Wellbeing in part because meaning in life (i.e., “life scheme”) was one of the most common dimensions in conceptualization and measurement of spirituality [55], however it is conceptually less clear how connection to nature would associate with self-efficacy.

In contrast, in Study 2, self-transcendence significantly mediated connection to nature and depression, perceived stress, positive states of mind, and satisfaction with life. The non-significant path from connection to nature to life satisfaction, which was contrary to previous research findings [2,6], became a significant indirect path through spirituality conceptualized as self-transcendence. This finding offers insight into the processes through which connection to nature may influence psychological wellbeing, suggesting that a sense of feeling connected to nature through transcendent experiences may predict greater life satisfaction.

### 4.3. Strengths

Strengths of this study include examination of mindfulness and spirituality simultaneously in a mediational model. Rather than using latent variables, we included separate indicators of mindfulness, spirituality, and psychological wellbeing, allowing greater specificity in identifying potential mechanisms linking connection to nature and psychological wellbeing.

### 4.4. Limitations and Future Directions

Our sample was predominantly White, female young adults who were never married and attended the same university, limiting the generalizability of our results. Replications of this study with more diverse samples are needed. We did not measure prior mindfulness experiences (meditation in particular) which has demonstrated a significant impact on the association between mindful observing and anxiety [35,81]. The use of different measures across two separate samples represents a limitation in interpreting the differing mediation patterns observed between Study 1 and Study 2. Future research could address this limitation by examining different measures of mindfulness and spirituality within a single sample. Moreover, we cannot make causal inferences from our mediational results because we utilized cross-sectional data. However, because connection to nature, mindfulness and spirituality are relatively trait-like, associations between these constructs and wellbeing may be stable over time in the absence of intervention. Future studies should consider experimental or longitudinal designs that test specific interventions known to promote these constructs (e.g., mindfulness practice).

## 5. Conclusions

In an era increasingly characterized by human disconnection from the natural world, it is critical more than ever to deepen our understanding of this connection, uncover how it may contribute to the wellbeing of humans and the planet, and develop effective responses to this growing disconnection. This research contributes to the conceptual understanding of how connection to nature may support wellbeing by examining mindfulness and spirituality—practices that invite us to cultivate presence, interconnectedness, and a deeper appreciation of life’s meaning—as potential explanatory mechanisms, aligning with calls to “better capture human wholeness” [58] (p. 5).

Realizing the interdependence between humans and the natural world has significant implications for individual and societal health and wellbeing. From policy, planning, and educational perspectives, it is important not only to provide opportunities for nature engagement but also to nurture a deeper sense of connection to nature and enhance the quality of people’s presence in it (e.g., engaging with nature through simple activities [5]). This shift in focus from “minutes in nature” to “quality of presence in nature” offers actionable strategies for professionals concerned with public health. For example, given the significant mediating role of mindful attention and awareness in reducing stress, nature-based health interventions may benefit from going beyond simply encouraging time spent outdoors to promoting mindful presence in natural environments. Additionally, because self-transcendence served as a mechanism linking connection to nature with positive psychological outcomes, public health interventions that provide awe-eliciting nature experiences and foster a sense of cosmic interconnectedness (e.g., public forest-bathing programs) may also be beneficial. This focus on the quality of presence in nature may foster mindfulness (i.e., being attentive, aware, and open to the present moment) and may facilitate spiritual experiences of belonging and relatedness (i.e., the feeling of being connected to a larger world that is home to both humans and beyond humans), thereby promoting human and planetary health.

## Figures and Tables

**Table 1:** Study 1 Participants’ demographic characteristics (*N* = 219).

Characteristics	*N* (%)
Age	
19–24 years old	214 (97.7)
Older than 24 years old	5 (2.3)
Gender	
Woman	152 (69.4)
Man	67 (30.6)
Grade	
Freshman	31 (14.2)
Sophomore	69 (31.5)
Junior	67 (30.6)
Senior	52 (23.7)
Race/Ethnicity	
Asian	20 (9.1)
Black or African American	4 (1.8)
Native Hawaiian or Pacific Islander	1 (0.5)
White	184 (84)
Other	10 (4.6)
Marital Status	
Married	4 (1.8)
Divorced	1 (0.5)
Never Married	214 (97.7)

**Table 2:** Study 1 Pearson’s correlation matrix for connection to nature, mindfulness, spirituality, and psychological wellbeing (*N* = 218).

Variables		1	2	3	4	5	6	7	8	9	10	11
Connection to Nature												
1.	CNS	--										
Mindfulness												
2.	Mindful Attention	0.19 **	--									
3.	Mindful Awareness	0.29 **	0.29 **	--								
4.	Mindful Acceptance	−0.03	0.52 **	0.02	--							
Spirituality												
5.	Self-Efficacy	0.02	0.36 **	0.21 **	0.44 **	--						
6.	Life Scheme	−0.06	0.30 **	0.16 *	0.41 **	0.62 **	--					
Psychological Wellbeing												
7.	Depression	0.04	−0.24 **	−0.25 **	−0.32 **	−0.54 **	−0.54 **	--				
8.	Anxiety	0.04	−0.44 **	−0.06	−0.49 **	−0.52 **	−0.35 **	0.48 **	--			
9.	Perceived Stress	0.04	−0.43 **	−0.08	−0.49 **	−0.54 **	−0.41 **	0.51 **	0.64 **	--		
10.	Life Satisfaction	−0.04	0.26 **	0.17 *	0.40 **	0.54 **	0.60 **	−0.57 **	−0.46 **	−0.57 **	--	
11.	Positive State of Mind	0.07	0.33 **	0.29 **	0.30 **	0.55 **	0.47 **	−0.68 **	−0.57 **	−0.57 **	0.53 **	--
M		3.56	3.59	3.61	2.68	3.85	3.60	1.60	2.23	3.03	5.01	3.20
SD		0.58	0.66	0.50	0.72	0.76	1.01	0.49	0.57	0.61	1.24	0.57
α		0.82	0.84	0.77	0.88	0.83	0.91	0.72	0.83	0.85	0.89	0.83

Note. * *p* < 0.05; ** *p* < 0.01.

**Table 3:** Study 2 Participants’ demographic characteristics (*N* = 180).

Characteristics	*N* (%)
Age	
19–24 years old	174 (96.7)
Older than 24 years old	6 (3.3)
Gender	
Woman	135 (75)
Man	42 (23.3)
Non-Binary	2 (1.1)
Prefer not to answer	1 (0.6)
Grade	
Freshman	29 (16.1)
Sophomore	67 (37.2)
Junior	41 (22.8)
Senior	40 (22.2)
Graduate	3 (1.7)
Race/Ethnicity	
Asian	3 (1.7)
American Indian or Alaska Native	1 (0.6)
Black or African American	14 (7.8)
Native Hawaiian or Pacific Islander	1 (0.6)
White	139 (77.2)
Other	22 (12.2)
Marital Status	
Married	4 (2.2)
Divorced	1 (0.6)
Never Married	175 (97.2)

**Table 4:** Study 2 Pearson’s correlation matrix for connection to nature, mindfulness, spirituality, and psychological wellbeing (*N* = 180).

Variables		1	2	3	4	5	6	7	8	9	10	11	12
Connection to Nature													
1.	CNS	--											
Mindfulness													
2.	Observing	0.36 **	--										
3.	Describing	0.04	0.12	--									
4.	Acting with Awareness	−0.06	−0.01	0.34 **	--								
5.	Non-judging	−0.03	−0.14	0.52 **	0.51 **	--							
6.	Non-reactivity	0.05	0.27 **	0.22 **	0.10	0.26 **	--						
Spirituality													
7.	Self-Transcendence	0.28 **	0.17 *	0.23 **	0.01	0.06	0.10	--					
Psychological Wellbeing													
8.	Depression	0.00	−0.04	−0.23 **	−0.30 **	−0.46 **	−0.16 *	−0.22 **	--				
9.	Anxiety	0.17	0.18 *	−0.28 **	−0.51 **	−0.57 **	−0.29 **	0.05	0.52 **	--			
10.	Perceived Stress	0.07	0.06	−0.30 **	−0.50 **	−0.53 **	−0.32 **	−0.18 *	0.45 **	0.66 **	--		
11.	Life Satisfaction	0.07	0.03	0.33 **	0.28 **	0.43 **	0.26 **	0.41 **	−0.48 **	−0.33 **	−0.49 **	--	
12.	Positive State of Mind	0.02	0.11	0.35 **	0.47 **	0.44 **	0.22 **	0.26 **	−0.44 **	−0.44 **	−0.49 **	0.39 **	--
M		3.44	3.33	3.37	2.93	3.05	2.99	4.00	1.64	2.44	3.00	4.82	3.14
SD		0.67	0.61	0.68	0.67	0.79	0.59	0.63	0.50	0.59	0.62	1.29	0.54
*α*		0.85	0.75	0.86	0.84	0.88	0.71	0.86	0.72	0.81	0.85	0.88	0.77

Note. * *p* < 0.05; ** *p* < 0.01.

## Data Availability

The data supporting the findings of this study were collected for the purpose of this research. Due to confidentiality and privacy considerations, the data are not publicly available. De-identified data are available from the corresponding author upon request.
